# Myokines in Response to a Tournament Season among Young Tennis Players

**DOI:** 10.1155/2016/1460892

**Published:** 2016-08-29

**Authors:** K. Witek, P. Żurek, P. Zmijewski, J. Jaworska, P. Lipińska, A. Dzedzej-Gmiat, J. Antosiewicz, E. Ziemann

**Affiliations:** ^1^Department of Biochemistry, Institute of Sport, Warsaw, Poland; ^2^Faculty of Physical Culture, University School of Physical Education Poznan, Gorzów Wielkopolski, Poland; ^3^Department of Physiology, Institute of Sport, Warsaw, Poland; ^4^Department of Physiology and Pharmacology, Gdansk University of Physical Education and Sport, Gdansk, Poland; ^5^Department of Biomechanics, Institute of Sport, Warsaw, Poland; ^6^Department of Bioenergetics and Physiology of Exercise, Medical University of Gdansk, Gdansk, Poland

## Abstract

The study investigated changes in myokines, heat shock proteins, and growth factors in highly ranked, young, male tennis players in response to physical workload during the competitive season and their potential correlations with match scores. Blood collections were carried out at the beginning, the midpoint, and the end of the tournament season. Data analysis revealed a significant increase in interleukin 6 and its inverse correlation with the number of lost games (*r* = −0.45; 90% CI −0.06 to 0.77). Neither the irisin nor BDNF level changed notably, yet delta changes of irisin across the season significantly correlated with the number of games won. The concentration of HSP27 recorded a small increase (31.2%; 90% CI 10.7 to 55.5, most likely). A negative correlation was noted between IGF-1 and HSP27 concentration at baseline (−0.70 very high; 90% CI −0.89 to −0.31, very likely). At the end of the season IGF-1 correlated positively with the number of games won (*r* = 0.37 moderate, 90% CI −0.16 to 0.73, likely) but negatively with the number of games lost (*r* = −0.39, 90% CI −0.14 to −0.74, likely). In conclusion our data indicated that Il-6, irisin, and growth factor IGF-1 may modify overall performance during a long lasting season, expressed in the amount of games won or lost.

## 1. Introduction

During a competitive season, professional tennis players experience mental and physical stress. Monitoring stress-inducing and manifesting factors is challenging; in research it is most often attempted during practice [1, 2] or a simulated tennis tournament [3]. Observations regarding the effects of the whole competitive season on the psychological and physical response are thus limited. The previous study revealed that during a tournament season most of the tennis players exhibited an elevated concentration of proinflammatory cytokines such as tumour necrosis factor (TNF*α*) or interleukin-1beta (IL-1*β*) [4, 5], which to some extent correlated inversely with the synthesis of heat shock proteins (HSP) [6]. Some of them, such as HSP27 and HSP70, were described as important for the repair and stabilization of stressed and damaged proteins [7]. Miller-Graziano et al. [8] stated that HSP27 belongs to a new group of “antidanger signals” that play a direct role in protecting against oxidative stress induced by exercise. Moreover, an elevated concentration of HSP70 is considered as a novel fatigue-signalling factor, sent from the immune system to the brain [9].

With regard to competitive sports, the focus of researchers and practitioners is on diagnosing and detecting signs of excessive mental and physical overload, associated with training sessions and competitions. It is particularly difficult or even impossible to measure physical workload among a group of tennis players, who train and plan their tournament seasons individually. In addition, monitoring of training loads in tennis is very demanding due to many variables that need to be accounted for undefined duration of activity and rest, number of games, matches, and tournaments played and unpredictable weather conditions throughout. These conditions make it difficult to establish a one model of periodized training plan. Moreover, altogether these factors may disturb the proper balance between anabolic and catabolic processes, which is crucial for adaptation processes.

The defensive response depends on the synthesis of anabolic agents such as insulin growth factor IGF-1, proteins binding, and regulating its concentrations (IGF-BP) or myokines, which have an anti-inflammatory effect, regulate energy metabolism [10], and stimulate other tissues to synthesise proteins responsible for cognitive functions [11]. Similarly to other disciplines, in tennis the processes of perception and decision-making are very exhausting both mentally and in terms of energy expenditure [12].

Two particularly important myokines have been described: interleukin 6 (IL-6) and the newly discovered irisin [13]. IL-6 was originally classified as a prototypical proinflammatory cytokine, while later anti-inflammatory properties were also described [14, 15]. Irisin is secreted into the circulation following proteolytic cleavage from its cellular form, fibronectin-type III domain-containing 5 (FNDC5), in response to exercise [13]. Some data have shown that irisin is also secreted by adipose tissue [16, 17]. Its ability to increase the metabolic rate as well as effectiveness in enhancing the energy expenditure appears to be potentially therapeutic for obesity [18]. Irisin is also of interest to sport scientists. Daskalopoulou et al. [19] noted a correlation between irisin and intensity of exercise among young athletes. The findings of Nygaard et al. [20] underlined the relationships between single sessions of endurance and strength training and irisin concentration. At the same time, another study suggested that irisin may be a biomarker of muscle damage or act as a protective agent [21]. Furthermore, irisin has been shown to increase the expression of peroxisome proliferator-activated receptor *γ* coactivator-1*α* (PGC-1*α*), which plays an important role in the expression of brain-derived neurotrophic factor (BDNF) [11], which is relevant for brain health and development as well as cognitive functions [22]. In light of these developments, the aim of this study was to evaluate the influence of the whole tournament workload on blood myokine IL-6 and irisin in elite young tennis players. An additional purpose of the study was to reexamine the roles of BDNF, HSP27, and HSP70 in development of overreaching syndrome.

## 2. Methods 

### 2.1. Overview

In this follow-up study, blood from young male tennis players was analysed on three occasions within the competitive season to evaluate the cumulative effects of match playing and its scores on selected biochemical indices. The study was conducted in the 2014 tournament season.

### 2.2. Subjects

**Figure 1:**
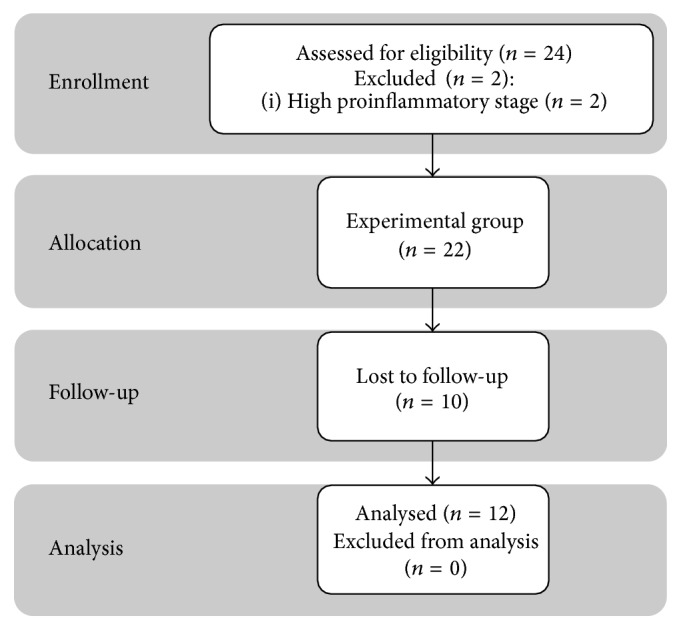
The schedule of examinations.

Highly ranked, national-level, young, male tennis players (age 16 ± 2 years, singles national ranking 1–30) took part in the experiment. Overall, 12 players left the study at the beginning due to either high concentrations of proinflammatory markers (*n* = 2), injury (*n* = 4), fear of blood sampling/fear of weakening physical ability (*n* = 2), or cancelled participation in selected tournaments (*n* = 4), leaving 24 participants (Figure 1). Blood collection was carried out at the beginning of the tournament season (January), at its midpoint (May), and at the end (September). To examine the physical workload, all games of the participating players were recorded during this period using an online system provided by the Polish Tennis Association (PZA).

The examination was officially approved by the Bioethical Committee of the Regional Medical Society in Gdansk (KB-26/14) according to the Declaration of Helsinki. Participation had to be approved by written consent from the tennis players' parents.

### 2.3. Blood Sampling and Cytokine Analysis

Blood samples were taken from an antecubital vein into single-use containers with an anticoagulant (EDTAK_2_). After collection, the samples were immediately stored at a temperature of 4°C. Within 20 minutes, they were centrifuged at 3000 g and 4°C for 10 min. Aliquots of the plasma were stored at −80°C. The blood was collected at rest, in the morning hours 7:30–8:00 a.m. in fasting state.

Serum (IL-6), interleukin-10 (IL-10), and TNF*α* concentrations were determined via enzyme immunoassay methods using commercial kits (R&D Systems, USA). The detection limits for TNF*α*, IL-6, and IL-10 were 0.039, 0.500, and 0.038 pg·mL^−1^, respectively. The average intra-assay CV was <8.0% for all cytokines.

Quantification of serum irisin was based on a competitive enzyme immunoassay and the assay kits purchased from Phoenix Pharmaceuticals Inc. (EK 067-16). The intra-assay coefficients of variability (CVs) and inter-assay CVs reported by the manufacturer were 4%–6% and 8%–10%, respectively.

Serum brain-derived neurotrophic factor was also detected using sandwich ELISA according to the manufacturers' instructions (R&D Systems, USA; DY248). The detection limit for BDNF was 15 pg·mL^−1^. Values are expressed as ng·mL^−1^. Based on our previous experiences and Maffioletti's recommendation, 1-hour clotting duration for a correct serum BDNF dosage was applied [23].

Serum heat shock proteins HSP27 and HSP70 were evaluated using a Calbiochem ELISA kit (USA) and Stressgen kit (USA). Detection limits were 0.2 ng·mL^−1^, and the intra-assay coefficient of variation for the kits was <5%.

Additionally, exercise-induced changes in plasma volume during the whole period of the investigation were calculated using the formula developed by van Beaumont et al. [24]. Thus, all myokines were recalculated according to changes in plasma volume using the formula proposed by Berthoin et al. [25].

### 2.4. Statistical Analysis

Measures related to blood parameters were analysed in a spreadsheet for a post-only crossover trial [26], and the effects were interpreted using magnitude-based inferences [27]. All data were log-transformed to reduce bias arising from the error nonuniformity. Means of the score changes, standard deviations of the score changes, and effects (differences in the changes of the means and their certainty limits) were backtransformed to percentage units. To improve the precision of estimates, the mean changes were adjusted to the log-transformed baseline mean. Magnitudes of the effects were also evaluated with the log-transformed data by standardizing with the standard deviation of the baseline values. Threshold values for assessing magnitudes of standardized effects were 0.20, 0.60, 1.2, and 2.0 for small, moderate, large, and very large, respectively. Uncertainty in each effect was expressed as a 90% confidence limit as well as a probability of the true effect being substantially positive (an increase) or negative (a decrease). These probabilities were used to make a qualitative, probabilistic, nonclinical inference about the true effect: if the probability of the effect being a substantial increase or a substantial decrease was >5% in both cases (equivalent of 90% confidence interval overlapping thresholds for a substantial increase and decrease), the effect was reported as unclear; otherwise, it was considered clear and assigned the relevant magnitude value, with the qualitative probability of the true effect being a substantial increase, substantial decrease, or a trivial difference (whichever outcome had the largest probability). The following scale for interpreting the probabilities was used: 25–75%, possible; 75–95%, likely; 95–99.5%, very likely; >99.5%, most likely. This study involved the assessment of substantial changes in nine measures. To maintain an overall error rate of <5% for declaring one or more changes to have opposite magnitudes (a substantial decrease instead of an increase, and vice versa), the effects were also evaluated as clear or unclear with a threshold of 5%/5 (1%), equivalent to consideration of overlap of substantial values with a 98% confidence interval (CI).

Relationships between changes in blood parameters across the tournament season against the number of performed games (all, won, and lost) were also calculated using Pearson correlation coefficients. Outcomes were expressed as values with 90% confidence intervals [28]. The usual scale for correlation coefficients (0.1, 0.3, 0.5, 0.7, and 0.9 for low, moderate, high, very high, and nearly perfect, resp.) was used.

## 3. Results

Obtained data of blood collections are presented in Table 1. Data show changes in the mean values of the effect induced by the workload applied during the tournament season and the magnitudes of the recorded shifts. The online system provided by Polish Tennis Association summarized all the games in this evaluated period. The average number of all games was 42 (±17) and of won and lost games was 26 (±15) and 16 (±5), respectively. The total number of matches included singles as well as doubles games. The more games the players won, the more they performed (0.97; 90% CI: 0.91 to 0.99). The physical workload experienced across the whole tournament season (training and tournaments) elicited a large and very large, clear increase in the IL-6 concentration (in the middle of the season and after the whole season, resp.). A moderate clear increase in anti-inflammatory interleukin (IL-10) was recorded but only in the middle of the season. Among HSP proteins a small clear and very likely increase was noted in HSP70 concentrations, whereas HSP27 elevated in smaller range (likely). All of these effects were still clear at the 98% CI level. The tournament season had no influence on proinflammatory level of TNF*α*. The moderate possible decrease in irisin concentration was noted in the middle of the season and trivial, but also possible decrease, at the end of the season. The other effects among measured blood parameters were only likely small and unclear.

**Figure 2:**
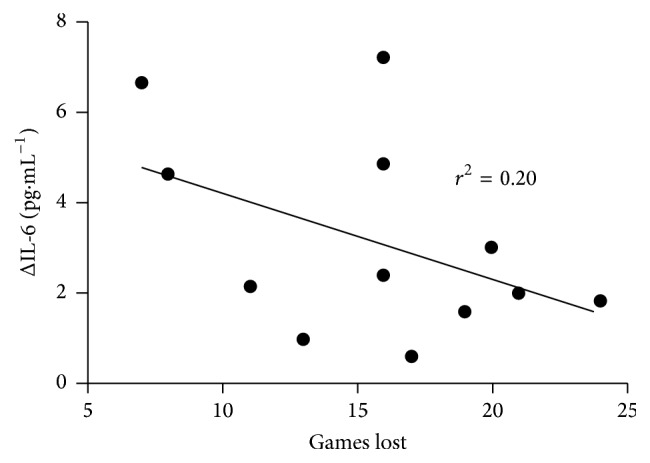
The relationship between delta changes in IL-6 concentration and number of lost games (*r* = −0.45, 90% CI −0.06 to 0.77, moderate, likely).

**Figure 3:**
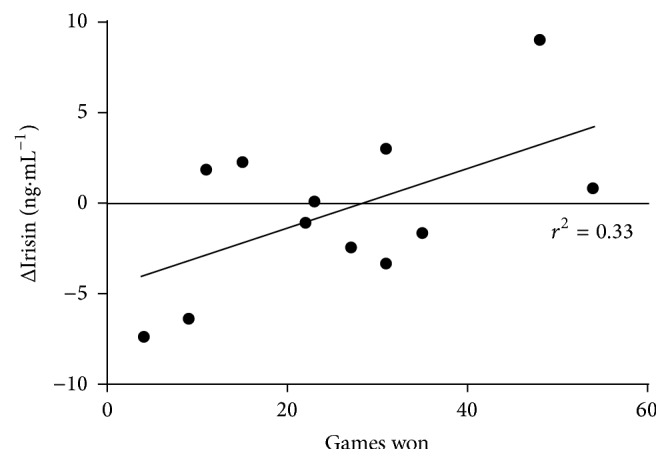
The correlation between delta changes in irisin concentration and number of won games (*r* = 0.57; 90% CI 0.01 to 0.83, high, very likely).

Calculation of relationships between changes in blood parameters and number of lost games showed negative, likely moderate to high correlations in changes in IL-6 and in IGFBP-3 (*r* = −0.45, 90% CI −0.06 to 0.77 (Figure 2); −0.43; 90% CI −0.77 to 0.09, resp.), while a likely positive high correlation was observed for changes in BDNF (0.49; 90% CI: 0.0 to 0.8). Although the tournament season was not significantly associated with irisin concentration, its delta changes across the season significantly correlated with the number of won games (likely moderate 0.45; 90% CI: −0.06 to 0.78, Figure 3), and the determination factor equalled 0.20. Among anabolic indicators delta changes of IGFBP-3 inversely and highly corresponded to the number of played games (−0.53; 90% CI −0.81 to 0.04). At the end of the season the IGF-1 level correlated positively with the number of games won (*r* = 0.37 moderate, 90% CI −0.16 to 0.73, likely) but negatively with the number of games lost (*r* = −0.39, 90% CI −0.14 to 0.74, likely).

## 4. Discussion

The main finding of the study is that the physical workload during the whole tournament season led to an elevated concentration of myokine IL-6 and a slight decrease in irisin concentration. It is worth noting that the rate of changes of IL-6 concentrations is inversely correlated with the number of games lost. Previous studies reported that IL-6 increases exponentially during a physical effort in relation to the intensity and duration of exercise, the mass of working muscles, and the individual's endurance capacity [29, 30]. Moreover, the biological role of IL-6 was described as an important energetic sensor, suggesting that muscle-liver crosstalk is mediated via IL-6 in regulating plasma glucose levels through endogenous glucose production during exercise [31]. In young adolescents a negative correlation of the amount of physical activity and plasma IL-6 concentration was found [32]. The significant shift of IL-6, which was observed at the end of the tournament season, indicates that those players who were characterized by greater changes in IL-6 concentration made fewer mistakes during the games. We can speculate that the rise of IL-6 can be treated as the anti-inflammatory response, which was supported by the increase of IL-10. Both allowed our players to avoid injuries or even an overreaching syndrome. Our results correspond with Halson's hypothesis, showing that reductions in IL-6 may lead to an altered metabolism of carbohydrate and fatty acids in the formation of ATP within skeletal muscle and induce the immune system dysfunction [33, 34]. Moreover, the latest paper by Wojewoda and coworkers revealed that IL-6 could be involved in the regulation of a moderate-intensity training-induced enhancement of muscle oxidative phosphorylation activity in locomotors skeletal muscles [35]. These findings let us suggest that the tennis players characterized by the elevated blood level of IL-6 had been well adapted to the long lasting season workload and had achieved better scores in tournaments. Still, in our group enhanced synthesis of HSP70 at the end of the season was recorded. The obtained data confirm the previous observation [6] that a physical and mental workload leads to HSP70 production. The relationship between the rise of IL-6 and HSP70 was not significant but demonstrated that an increase of IL-6 inhibits HSP70 synthesis. The lack of statistical significance may be due to the small number of participants and should be verified in further studies.

One of the factors, which can influence obtained data, is the number of tournaments and games played by each player. For example, in our group of tennis players the total number of tournaments and games was lower than the number performed by the best players according to the International Tennis Federation. The best juniors played throughout season 91 (no. 1) and 90 (no. 2) singles and doubles matches. The average top 10 juniors played 81 games per season. The best player under 16 (no. 1) played 43 matches, but no. 2 played as many as 98 matches. The average top 10 played 58 matches per season. In our group the tennis players who played more won more games. On one hand, it seems to be logical that the more they played, the more experience they gained; but on the other hand, the more they played, the more physical work they performed and they could feel more exhausted. In training and competition it is demanding to maintain a balance between anabolic and catabolic response. Thus, we evaluated the influence of competitive season on IGF-1 and IGFBP-3. The latter belongs to the family of binding proteins which bind IGF-1 in the blood and in the extracellular matrix [36]. IGF-1 bound by IGFBP-3 cannot interact with a receptor, which inhibits its effect on gene expression [37]. At the same time, IGF-1 bound by IGFBP-3 is protected against prompt removal from the blood circulation; thus increase of its concentration may potentiate IGF-1 effects [38].

In our study we observed both proteins to have increased during the season; however, the increase of IGFBP-3 was much more pronounced, and it can be considered as an adaptive response. In addition, among our tennis players a significant, inverse correlation between delta change of IGFBP-3 and amount of lost games was observed. Previously, in endurance sports, a fatigue-dependent course was observed for IGF-1, but this kind of change was not observed as a cumulative effect of sports game training. It was proposed that because IGF-1 and IGFBP-3 are functionally connected and mainly represent the metabolic aspect of fatigue, a different kind of tennis match and training as compared to endurance training may therefore partly explain the smaller effect sizes of fatigue-induced changes [39]. Elevated concentrations of IGF-1 and IGFBP-3 were noted in the high level training group of young volleyball players after 18 months of intensive training compared to controls [40]. However, other patterns could also be observed. It was proposed that the state of a decrease in IGF-1 accompanied by an increase in IGF-BP3 could indicate a state of glucose austerity after depletion of carbohydrate stores due to endurance training [41]. In young boxers, IGF-1 and IGF-BP3 did not change significantly after a 5-week period of intense training but greatly increased after one week of tapering [42], suggesting an adaptive response. IGF-1 and IGF-1/cortisol ratio were found to be sensitive markers of training load and physical performance variations [42]. Moreover, in young individuals, a positive relationship was found between IGF-1 concentration and physical performance [43].

In our study the second myokine and irisin was considered as an important factor, which may not only regulate metabolism but also stimulate cognitive functions [11]. In contrast to our expectation, the effect of the tournament season caused a trivial decrease in the concentration of irisin and consequently BDNF level. Interestingly, a positive correlation was noted between the rate of change of irisin and number of won games. These data support the concept that irisin may be a link connecting function of skeletal muscle and brain. On the other hand, no changes were recorded in BDNF concentration. It has been also shown that serum BDNF levels reflect the BDNF concentration in the brain; therefore, measurements of the serum BDNF concentration can be used to monitor its changes in the brain [44]. In another study, both acute aerobic and anaerobic activity elevated serum BDNF in athletes. It was suggested that long-term habitual exercise is associated with lower peripheral BDNF and better intermediate memory [45]. However, acute forms of intensive activity, either aerobic or anaerobic, are able to elevate serum BDNF level in both sedentary persons and athletes [45]. It was also reported that endurance training of moderate intensity increases both basal and end-exercise BDNF levels in young healthy men [46]. These results suggest a possible relationship between irisin and cognitive function among our tennis players. One of the factors which can modulate BDNF synthesis is the proinflammatory cytokine TNF*α* [47]. It is also known that other factors, which induce inflammation, contribute to reducing the BDNF concentration [48, 49]. Also, some types of athletic activities like heading a ball could increase the BDNF concentration in the blood, which is related to a microtrauma of the brain tissue [47], but this type of movement act is not typical for a tennis activity and has a rather minor contribution. In our tennis players we did not observe any significant rise in TNF*α*, neither in the middle, nor at the end of the tournament season. Obtained serum BDNF concentrations in our group exhibited elevated values in comparison to the recommended ones [48].

## 5. Conclusion

To authors knowledge this is one of the first study presenting changes of broad biochemical and immunological indices within competitive season in tennis players. Despite being partially limited by the small number of subjects and lack of monitoring of training workload of each player, the report provides selected reference data.

Present data demonstrating that myokines IL-6 and irisin and IGF-1 and IGFBP-3 could be useful markers in monitoring tennis workload and exercise adaptations. The observed changes indicate these factors contribute to a defence mechanism and have an impact on the cognitive functions, which enables players to make better, more strategic decisions during game.

## Figures and Tables

**Table 1:** The immunological response induced by physical workload during tournament season. Measures related to blood parameters at baseline and changes in the measures in the middle of season and at the end of the season in young tennis players (*n* = 12).

	Baseline mean ± SD	Middle season change		After season change	
		Mean; CI	Inference	Mean; CI	Inference
TNF*α* (pg·mL^−1^)	1.15 ± 0.44	−8.8%; −21.6 to 6.0%	Trivial↓^*∗*^	0.2%; −10.9 to 12.7%	Unclear
IL-6 (pg·mL^−1^)	1.12 ± 0.57	92.5%; 64.1 to 126%	Large↑^*∗∗∗∗*^	280%; 182 to 413%	Very large↑^*∗∗∗∗*^
IL-10 (pg·mL^−1^)	0.58 ± 0.21	73%; 11.3 to 169%	Moderate↑^*∗∗*^	38%; −24.4 to 152%	Small↑^*∗*^
HSP 70 (ng·mL^−1^)	0.18 ± 0.16	36.8%; 9.6 to 70.8%	Small↑^*∗*^	126%; 57.4 to 223%	Small↑^*∗∗∗*^
HSP 27 (ng·mL^−1^)	13.6 ± 7.11	5.9%; −6.9 to 20.6%	Trivial↑^*∗*^	31.2%; 12.6 to 52.8%	Small↑^*∗∗*^
IGF 1 (ng·mL^−1^)	228 ± 68	5.2%; −5.3 to 16.8%	Trivial↑^*∗*^	9.5%; −7.9 to 30.2%	Unclear
IGFBP-3 (ng·mL^−1^)	4137 ± 617	6.5%; 0.5 to 12.9%	Small↑^*∗∗*^	5.2%; −1.4 to 12.0%	Small↑^*∗*^
Irisin (ng·mL^−1^)	24.2 ± 22.5	−9.0%; −23 to 7.2%	Moderate↓^*∗*^	−2.1%; −11.8 to 8.6%	Trivial↓^*∗*^
BDNF (ng·mL^−1^)	50.9 ± 12.9	−14.7%; −38.5 to 18.2%	Unclear	−6.1%; −33.1 to 31.7%	Unclear

CI: 90% confidence interval.

↑: increase; ↓: decrease.

Likelihood that the true effect is substantial: ^*∗*^possible, ^*∗∗*^likely, ^*∗∗∗*^very likely, and ^*∗∗∗∗*^most likely.
